# Influence of Body Mass Index on Eating Habits and Food Choice Determinants Among Brazilian Women During the COVID-19 Pandemic

**DOI:** 10.3389/fnut.2021.664240

**Published:** 2021-07-12

**Authors:** Bruna Caruso Mazzolani, Fabiana Infante Smaira, Gabriel Perri Esteves, Heloísa C. Santo André, Milla Cordeiro Amarante, Daniela Castanho, Karen Campos, Fabiana Braga Benatti, Ana Jéssica Pinto, Hamilton Roschel, Bruno Gualano, Carolina Ferreira Nicoletti

**Affiliations:** ^1^Applied Physiology and Nutrition Research Group, Laboratory of Assessment and Conditioning in Rheumatology, Faculdade de Medicina FMUSP, Hospital das Clínicas HCFMUSP, Universidade de Sao Paulo, Sao Paulo, Brazil; ^2^School of Applied Sciences, State University of Campinas, Limeira, Brazil; ^3^Food Research Center, University of São Paulo, Sao Paulo, Brazil

**Keywords:** obesity, eating behavior, social isolation, confinement, SARS-CoV-2

## Abstract

Changes in emotional state due to the COVID-19 pandemic may potentially modify eating habits, which may differ as a function of body mass index (BMI). Using a self-reported, questionnaire-based survey we evaluated Brazilian women during the pandemic for: (i) the influence of BMI on changes in eating habits, food choice determinants, and psychological symptoms; (ii) associations between eating habits, food choice determinants and psychological symptoms. General characteristics, anthropometric data, eating habits before and during the pandemic, food choice determinants and psychological symptoms during the pandemic were collected between June and September, 2020. Participants (*n* = 1,183) were normal weight (60.4%), overweight (26.2%) and obese (13.4%). A higher frequency of “cooking” (72.3–77.6%, *p* = 0.004) and “use of delivery service” (29.8–48.8%, *p* < 0.001) was reported during, in comparison to before the pandemic. Additionally, a higher prevalence of “snacking” (57.1–63.8%, *p* = 0.005) and “eating at table” (78.5–82.7%, *p* < 0.001) was reported during the pandemic, while the number of participants reporting “dieting” decreased (28.7–20.4%, *p* < 0.001). “Health”, “natural concerns” and “need and hunger” were less important determinants for participants with overweight/obesity compared to normal weight. Regression analysis indicated that *(i)* “health”, “natural concerns” and “affect regulation”; *(ii)* “health”, “pleasure”, “convenience”, and “natural concerns”; and *(iii)* “visual appeal” and “pleasure” were the food choice determinants more associated with eating habits among women with normal weight, overweight, and obesity, respectively. In conclusion, eating habits were modified during the pandemic despite BMI, whereas food choice determinants differed between overweight/obesity and normal weight women.

## Introduction

Quarantine and social isolation bring many challenges, including distancing of family and friends, loss of freedom, concern for one's health status, and boredom (1). To contain the COVID-19 pandemic, social distancing has been imposed worldwide. As a consequence, psychological symptoms, stress, irritability, insomnia, feelings of anger, and emotional exhaustion have been reported (1). These changes in emotional state can potentially modify lifestyle habits, including eating habits.

It has been suggested that changes to routine, such as those that occur during quarantines, can have a greater impact in women since they often hold more responsibilities regarding family food choices (2). In addition, women tend to eat more in stressful situations when compared to men (3). A study evaluating choices, behaviors and food preferences among women with normal weight, overweight or obesity showed that: (*i)* Taste is fundamental for food choice irrespective of BMI; (*ii)* Price was a greater determinant for food choice among women with overweight and obesity *vs*. normal weight; (*iii)* Health was an influential factor in food choice mostly among the participants with normal weight; and (*iv)* Women with overweight and obesity report liking a wider variety of foods, both healthy and less healthy, and mentioned using food more often as a mechanism to cope with periods of stress, depression or boredom, whereas their normal weight peers reported eating less or the same amount of food when facing negative emotions (4). These data collectively suggest that body mass index (BMI) plays an important role in food choices and preferences among women.

Eating habits and the complex process of food choices are driven by an interplay of physiological mechanisms, genetics, epigenetics, economic and behavioral factors, and organoleptic characteristics of foods (5, 6). The COVID-19 pandemic resulted in profound changes in women's daily routine that may potentially influence eating habits in unpredictable ways. Whether, and to what extent, BMI is associated with modifications in eating habits during the pandemic remains unknown.

The aims of this study were to evaluate: (i) the influence of BMI on changes in eating habits during the COVID-19 pandemic; (ii) the influence of BMI on food choice determinants and psychological symptoms during the COVID-19 quarantine and; (iii) associations between eating habits, food choice determinants, and psychological symptoms during the COVID-19 quarantine among Brazilian women.

## Methods

### Study Design and Participants

This is a cross-sectional, self-reported, questionnaire-based survey conducted between June and September 2020, a period during which a set of social distancing measures to contain the spread of COVID-19 were in place in Brazil. Participants were recruited through advertisements on social media platforms (Facebook®, WhatsApp®, Instagram®, Twitter®), press release, television, journals, and radio. Inclusion criteria were as follows: women aged ≥18 years, currently living in Brazil, with ability to read, and with internet access.

All participants completed an online survey on Google® Forms platform (Google® LLC, Menlo Park, CA, USA), which enquired about their demographic, socioeconomic, and anthropometric characteristics, psychological symptoms, lifestyle, and eating habits.

### Evaluation Tool

The online survey included questions categorized into the following sections: (*i)* participant characteristics (i.e., age, ethnicity, marital status, education level, smoking habits, and chronic medical conditions); (*ii)* anthropometric data (i.e., self-reported weight and height, which was then used to calculate BMI); (*iii)* eating habits quarantine (i.e., “cooking”, “participation in grocery shopping”, “use of delivery services”, “alcohol consumption”, “snacking”, “replacing main meals for snacks”, “eating at table”, “eating in front of television/tablet/cellphone”, and “dieting”). Participants were asked to self-report these eating habits as binary outcomes (i.e., “yes”, if participant reported a certain eating habit, or “no”, if participant reported the absence of a certain eating habit), both before (retrospective), and during (current) the quarantine. To the best of our knowledge, there is no validated instrument to directly assess eating habits available in the literature. Therefore, we based our questions on the Dietary Guidelines for the Brazilian Population (7), to better capture the most relevant eating habits of our sample.

Participants also completed previously validated tools commonly used to assess food choice determinants and psychological symptoms. These included: The Brazilian Portuguese version of The Eating Motivation Survey (TEMS) (8, 9), which comprises 45 questions preceded by “I eat what I eat,…” and which is used to evaluate food choices determinants (i.e., “liking”, “health”, “natural concerns”, “need and hunger”, “habits”, “pleasure”, “convenience”, “weight control”, “sociability”, “traditional eating”, “price”, “visual appeal”, “affect regulation”, “social norms” and “social image”). The answers are given in a five-point scale ranging from 1 (“never”) to 5 (“always”), with higher scores representing a higher impact of a given food choice determinant. The Binge Eating Scale (BES) was used to evaluate symptoms of binge eating episodes (10, 11), with higher scores representing more symptoms of binge eating episodes (scores range from 0 to 46). The short version of the Disordered Eating Attitude Scale was employed to evaluate eating attitudes (12), with higher scores representing more dysfunctional eating attitudes (scores range from 17 to 75). The Depression Anxiety Stress Scale-21 (DASS-21) was used to assess depression, anxiety and stress symptoms (13, 14) with higher scores representing more symptoms (score range for: 0–28). The UCLA Loneliness Scale was used to assess perceived feelings of loneliness (15, 16), with higher scores representing more symptoms (score range for: 1–8). Importantly, these inventories are not validated for retrospective purposes; therefore, the data are reflective of “during COVID-19 quarantine” only.

### Data Privacy and Ethics Aspects

This study was approved by the local ethical committee and was conducted in accordance with the Helsinki declaration. Approved Informed Consent Form was signed digitally by all participants before initiating the survey.

### Statistical Analysis

Potential changes in eating habits due to COVID-19 quarantine (e.g., “cooking”, “participation in grocery shopping”, “snacking”, “replacing main meals for snacks”, “dieting” –dependent variables) were assessed by generalized estimating equations (GEE) model, based on the assumption of a binomial distribution, a logit link function, and an exchangeable working correlation, with group (normal weight, overweight, and obese) and time (before and during social distancing) as fixed factors and subjects as the random factor. The presence of outliers was tested using the Extreme Studentized Deviate method and normality was checked using the Shapiro-Wilk test for all continuous variables. The influence of BMI on food determinants, binge eating symptoms, eating attitude and psychological symptoms during the COVID-19 quarantine was assessed using a generalized estimating equation (GEE) model, based on the assumption of a normal distribution, an identity link function, and an exchangeable working correlation, with group (normal weight, overweight, and obese) included as the fixed factor. All GEE models were adjusted for age, education level, ethnicity, marital status, and number of comorbidities. Tukey's *post-hoc* test was used for multiple-comparison correction. Finally, associations between eating habits (independent variables) and determinants of food choices (dependent variables) were tested using linear regression models. All regression models were adjusted for age, BMI, educational level, ethnicity, marital status, and number of comorbidities. Data are presented as mean and 95% confidence interval (95% CI), absolute and relative frequency (n[%]) or β (95% CI). All analyses were performed using the statistical package SAS (version 9.4). The level of significance was set at *p* ≤ 0.05.

## Results

One thousand two hundred and forty-one participants completed the online survey; after removing duplicates and incomplete answers, 1,183 were included in the analysis. Age ranged between 18 and 72 years (34.56 [33.85, 35.27]). Most participants were white (77.8%), single (55.5%), and had a high education level (i.e. university level) (72.4%). According to BMI, 60.4% were classified as normal weight, 26.2% as overweight and 13.4% as obese. Age (*p* < 0.001), ethnicity (*p* = 0.012), marital status (*p* < 0.001), educational level (*p* = 0.007), and self-reported prevalence of comorbidities (all *p* < 0.050) were different between groups (Table 1).

**Table 1:** Participants' characteristics and eating habits before and during the COVID-19 quarantine among Brazilian women.

	**Total (*n* = 1,183)**	**Normal weight (*n* = 715)**	**Overweight (*n* = 310)**	**Obese (*n* = 158)**
Age (years)	34.56 (33.85, 35.27)	32.56 (31.67, 33.45)	37.81 (36.45, 39.16)^a^	37.34 (35.44, 39.25)^a^
BMI (kg/m^2^)	24.79 (24.51, 25.08)	21.68 (21.51, 21.85)	27.10 (26.84, 27.36)^a^	34.41 (34.06, 34.78)^a^^,^^b^
***Self-reported ethnicity***
White	921 (77.8%)	575 (80.6%)	232 (74.8%)	113 (71.5%)
Asian	41 (3.5%)	27 (3.8%)	9 (2.9%)	5 (3.2%)
Brown	159 (13.4%)	82 (11.5%)	47 (15.2%)	29 (18.4%)
Black	51 (4.3%)	23 (3.2%)	18 (5.8%)	10 (6.3%)
Indigenous	6 (0.5%)	2 (0.3%)	3 (1.0%)	1 (0.6%)
***Marital status***
Married	436 (36.9%)	219 (30.7%)	147 (47.5%)	70 (44,3%)
Single	657 (55.5%)	439 (63.4%)	132 (43.6%)	72 (46.2%)
Divorced	78 (6.6%)	40 (5.6%)	25 (8.1%)	13 (8.2%)
Widow	12 (1.0%)	6 (0.8%)	3 (1.0%)	3 (1.9%)
***Educational level***
Elementary school	12 (0.1%)	3 (0.4%)	5 (1.6%)	4 (2.6%)
High school degree	314 (26.5%)	193 (26.8%)	68 (21.9%)	53 (33.6%)
University degree	857 (72.4%)	518 (72.7%)	237 (76.5%)	101 (63.9%)
***Presence of chronic diseases***
Hypertension	50 (4.2%)	15 (2.1%)	13 (4.2%)	22 (13.9%)^a^^,^^b^
Diabetes mellitus	17 (1.4%)	4 (0.6%)	4 (1.3%)	9 (5.7%)^a^^,^^b^
Dyslipidemia	77 (6.5%)	32 (4.5%)	32 (10.3%)^a^	13 (8.2%)^a^
Thyroid disorders	109 (9.2%)	52 (7.3%)	36 (11.6%)^a^	21 (13.3%)^a^
Cardiovascular diseases	21 (1.8%)	7 (1.0%)	11 (3.5%)^a^	3 (1.9%)
Cancer	4 (0.3%)	1 (0.1%)	1 (0.3%)	2 (1.3%)
***Smoking habit***	63 (5.3%)	35 (4.9%)	21 (6.8%)	7 (4.4%)
***Eating habits before COVID-19 quarantine***
Participation in grocery shopping	880 (74.4%)	501 (70.1%)	248 (80.0%)^a^	131 (82.9%)^a^
Cooking	855 (72.3%)	497 (69.5%)	237 (76.5%)	121 (76.6%)
Use of delivery service	353 (29.8%)	216 (30.2%)	92 (29.7%)	45 (28.5%)
Eating at table	929 (78.5%)	567 (79.3%)	248 (80.0%)	114 (72.2%)
Eating in front of TV/tablet/cellphone	607 (57.1%)	358 (50.1%)	159 (51.3%)	90 (57.0%)
Replacing mean meals for snacks	400 (33.8%)	197 (27.6%)	125 (40.3%)^a^	78 (49.4%)^a^
Snacking	676 (57.1%)	393 (55.0%)	178 (57.4%)	105 (66.5%)
Dieting	340 (28.7%)	160 (22.4%)	116 (37.4%)^a^	64 (40.5%)^a^
Alcohol consumption	821 (69.4%)	506 (70.8%)	205 (66.1%)	110 (69.6%)
***Eating habits during COVID-19 quarantine***
Participation in grocery shopping	811 (68.6%)^*^	472 (66.0%)	220 (71.0%)	119 (75.3%)
Cooking	918 (77.6%)^*^	543 (75.9%)	249 (80.3%)	126 (79.7%)
Use of delivery service	578 (48.9%)^*^	343 (48.0%)	148 (47.7%)	87 (55.1%)
Eating at table	978 (82.7%)^*^	589 (82.4%)	261 (84.2%)	128 (81.0%)
Eating in front of TV/tablet/cellphone	612 (51.7%)	359 (50.2%)	162 (52.3%)	91 (57.6%)
Replacing mean meals for snacks	401 (33.9%)	224 (31.3%)	117 (37.4%)^a^	60 (38.0%)
Snacking	755 (63.8%)^*^	449 (62.8%)	203 (65.5%)	103 (65.2%)
Dieting	241 (20.4%)^*^	126 (17.6%)	72 (23.2%)	43 (27.2%)
Alcohol consumption	650 (54.9%)^*^	381 (53.3%)^*^	184 (59.4%)	85 (53.8%)

*Data are presented as mean and 95% confidence interval (95% CI) or absolute and relative frequency (n [%]). For binary variables frequency (n [%]) represents the presence of the habit*.

* *p <0.05 vs. before COVID-19 quarantine*.

a *p <0.05 vs. normal weight*;

b *p <0.05 vs. overweight. BMI, body mass index*.

Regarding the influence of BMI on changes in eating habits due to COVID-19, before quarantine, a greater number of overweight and obese women reported “dieting” when compared to the normal weight group (37.4 vs. 22.4%, *p* < 0.0001 and 40.5 vs. 22.4%, *p* < 0.0001). Similarly, overweight and obese reported “replacing main meals with snacks” more frequently than normal weight women (40.3 vs. 27.6%, *p* < 0.0001 and 49.4 vs. 27.6%, *p* < 0.001), as well as “participating in grocery shopping” (80.0 vs. 70.1%, *p* = 0.015 and 82.9 vs. 70.1%, *p* = 0.018). During quarantine, the overweight group reported “replacing main meals with snacks” more frequently than the normal weight group (37.4 vs. 31.3%, *p* = 0.038) (Table 1).

**Figure 1:**
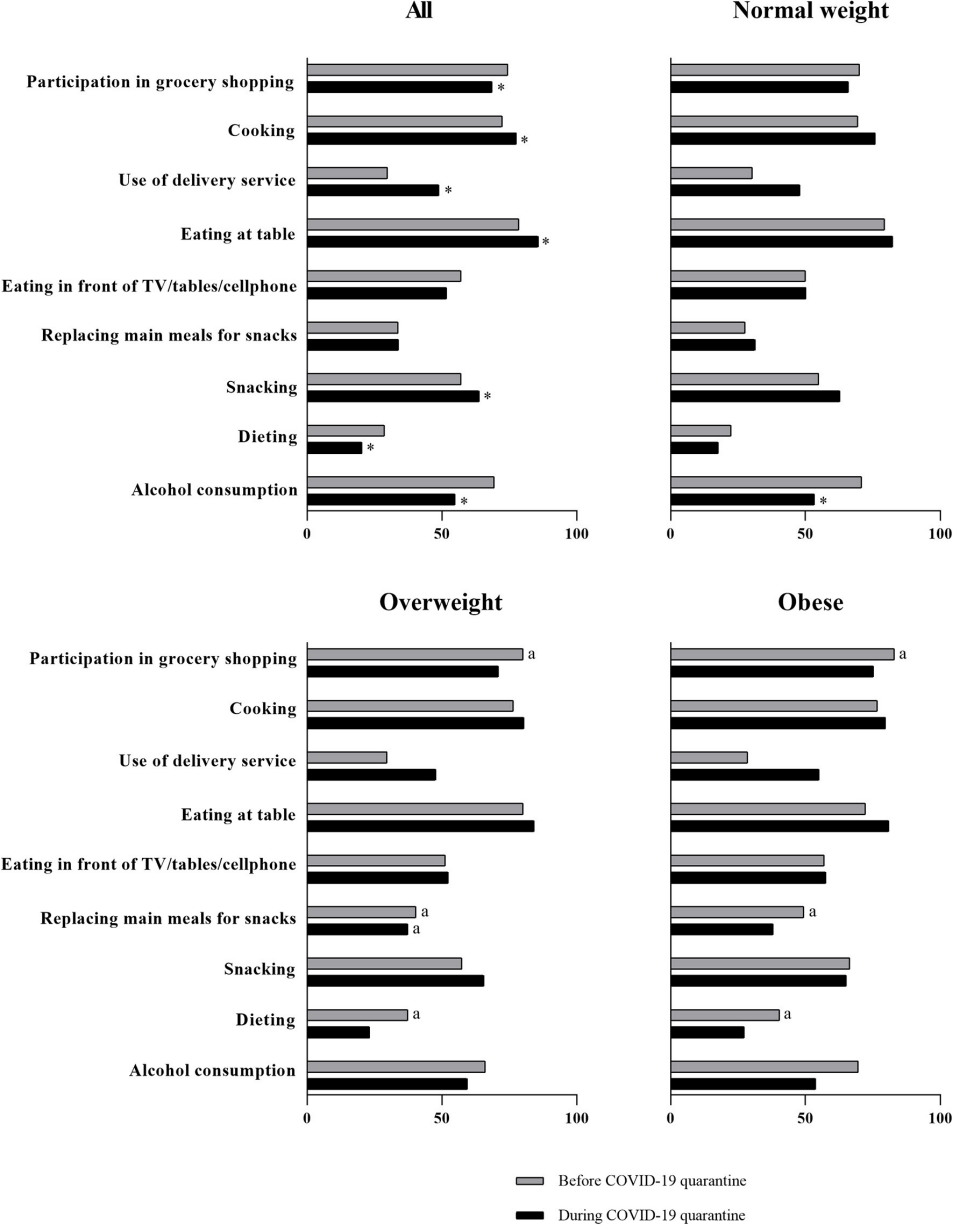
Changes in eating habits during the COVID-19 quarantine among Brazilian women. Data are presented as frequency (n [%]) for the presence of the habit; *p < 0.05 vs. before COVID-19 quarantine. ^a^*p* < 0.05 vs. normal weight.

When data were polled, significant changes from before, to during, quarantine were observed (Figure 1). “Participation in grocery shopping” reduced (74.4–68.6%, *p* < 0.001) during social distancing, whereas “cooking” (72.3–77.6%, *p* = 0.004) and “use of delivery service” (29.8–48.8%, *p* < 0.001) increased. The number of participants “eating at table” increased (78.5–82.7%, *p* < 0.001), but no changes were observed for “eating in front of TV/tablet/cellphone” (57.1–51.7%, *p* = 0.693). No changes were observed for the habit of “replacing main meals for snacks” (33.8–33.9%, *p* = 0.084), but there was an increase in the number of participants reporting the habit of “snacking” (57.1–63.8%, *p* = 0.005). The number of participants reporting the habit of “dieting” decreased (28.7–20.4%, *p* < 0.001). Also, the number of participants consuming alcohol beverages reduced during social distancing (69.4–54.9%, *p* < 0.001) (Table 1 and Figure 1).

Food choice determinants and psychological symptoms were different as a function of BMI. “Health” (overweight vs. normal weight, *p* < 0.0001; obese vs. normal weight, *p* < 0.0001), “natural concerns” (overweight vs. normal weight, *p* = 0.016; obese vs. normal weight, *p* < 0.0001), “need and hunger” (overweight vs. normal weight, *p* < 0.0001; obese vs. normal weight, *p* < 0.0001) and “habits” (overweight vs. normal weight, *p* = 0.006; obese vs. normal weight, *p* < 0.0001) were less important determinants for participants with overweight and obesity compared to those with normal weight (Table 2). In contrast, “affect regulation” was a more important determinant for participants with overweight and obesity compared to those with normal weight (overweight vs. normal weight, *p* < 0.0001; obese vs. normal weight, *p* < 0.0001), “health” (*p* = 0.003), “natural concerns” (*p* = 0.02) and “affect regulation” (*p* = 0.0004) were less important for participants with overweight than obesity (Table 2). Disordered eating attitudes (overweight vs. normal weight, *p* < 0.0001; obese vs. normal weight, *p* < 0.0001) and symptoms of binge eating episodes (overweight vs. normal weight, *p* = 0.038; obese vs. normal weight, *p* = 0.0005), depression (overweight vs. normal weight, *p* = 0.038; obese vs. normal weight, *p* = 0.0005) and anxiety (overweight vs. normal weight, *p* = 0.049; obese vs. normal weight, *p* < 0.0001) were more prevalent in participants with overweight and obesity, compared to those with normal weight (Table 2). Moreover, disordered eating attitudes (*p* = 0.001) and symptoms of binge eating episodes (*p* < 0.0001), and anxiety (*p* = 0.028) were more prevalent in participants with obesity than in those with overweight (Table 2). Stress was more prevalent among participants with obesity compared to those with normal weight (*p* = 0.0004; Table 2). “Liking”, “need and hunger”, and “habits” were the most commonly reported determinants of food choices for all groups (Table 2).

**Table 2:** Food choice determinants and psychological symptoms during the COVID-19 quarantine among Brazilian women.

	**Normal weight**	**Overweight**	**Obese**
*TEMS (I eat what I eat …)*			
Liking	3.00 (2.95, 3.05)	2.92 (2.84, 3.00)	2.93 (2.82, 3.04)
Health	2.55 (2.48, 2.61)	2.25 (2.15, 2.35)^a^	1.96 (1.82, 2.10)^a^^,^^b^
Natural concerns	1.95 (1.88, 2.02)	1.79 (1.68, 1.90)^a^	1.50 (1.35, 1.65)^a^^,^^b^
Need and hunger	2.81 (2.75, 2.86)	2.53 (2.45, 2.61)^a^	2.38 (2.26, 2.49)^a^
Habits	2.75 (2.69, 2.81)	2.58 (2.49, 2.67) ^a^	2.43 (2.30, 2.55)^a^
Pleasure	2.02 (1.96, 2.08)	2.13 (2.04, 2.23)	2.18 (2.05, 2.32)
Convenience	1.95 (1.89, 2.00)	1.88 (1.78, 1.98)	1.93 (1.80, 2.05)
Weight control	1.46 (1.39, 1.52)	1.56 (1.46, 1.65)	1.41 (1.29, 1.55)
Sociability	1.65 (1.58, 1.72)	1.72 (1.61, 1.82)	1.57 (1.42, 1.71)
Traditional eating	1.73 (1.67, 1.80)	1.74 (1.65, 1.84)	1.71 (1.57, 1.84)
Price	1.49 (1.42, 1.56)	1.37 (1.27, 1.47)	1.50 (1.36, 1.64)
Visual appeal	1.08 (1.02, 1.14)	1.18 (1.09, 1.27)	1.20 (1.07, 1.32)
Affect regulation	1.01 (0.94, 1.08)	1.30 (1.20, 1.41)^a^	1.65 (1.51, 1.80)^a^^,^^b^
Social norms	1.09 (1.04, 1.15)	1.04 (0.95, 1.13)	1.18 (1.06, 1.30)
Social image	0.41 (0.36, 0.45)	0.46 (0.40, 0.53)	0.48 (0.39, 0.57)
*DEAS score*	27.55 (26.81, 28.29)	31.36 (30.25, 32.48)^a^	35.28 (33.71, 36.82)^a^^,^^b^
*BES score*	7.01 (6.49, 7.52)	11.51 (10.73, 12.30)^a^	15.41 (14.32, 16.51)^a^^,^^b^
*Depression symptoms score*	5.72 (5.32, 6.12)	6.16 (5.55, 6.76)^a^	7.30 (6.46, 8.14)^a^
*Anxiety symptoms score*	3.82 (3.49, 4.16)	4.32 (3.81, 4.83)^a^	5.73 (5.02, 6.45)^a^^,^^b^
*Stress symptoms score*	7.74 (7.34, 8.13)	8.28 (7.68, 8.88)^a^	9.30 (8.47, 10.14)^a^
*Loneliness Scale score*	30.81 (29.88, 31.74)	31.38 (29.97, 32.79)	34.11 (32.14, 36.09)^a^

*Data presented as mean and 95% confidence interval (95% CI)*.

a *p <0.05 vs. normal weight*;

b *p <0.05 vs. overweight*.

*DEAS, Disordered Eating Attitude Scale; BES, Binge Eating Scale*.

*For The Eating Motivation Survey (TEMS), the sub-items for assessing motives for eating behavior were introduced by the following item stem “I eat because …” or by “I select certain foods because …” and answers were given on a five-point rating scale from 1 “never” to 5 “always”*.

**Figure 2:**
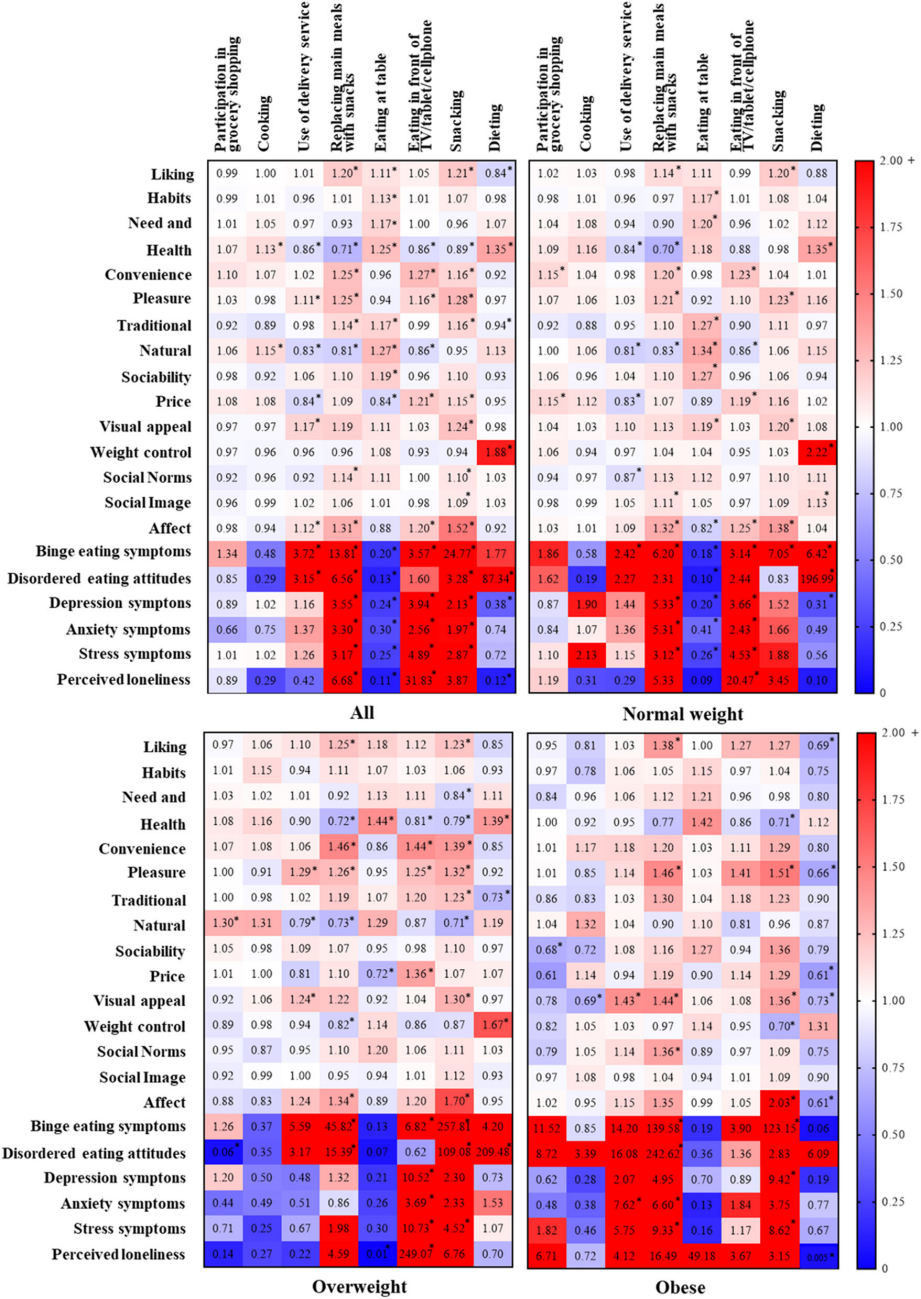
Associations between eating habits, food choice determinants, and psychological symptoms during the COVID-19 quarantine among Brazilian women. Data are presented as as odds ratio (OR). *p < 0.05.

Linear regression models showed that “health”, “natural concerns” and “affect regulation” were the food choice determinants associated with a higher number of eating habits (≥3) among women with normal weight (Figure 2). “Health”, “pleasure”, “convenience”, and “natural concerns” were the determinants associated with a higher number of eating habits among women with overweight, whereas “visual appeal” and “pleasure” were those associated with a higher number of eating habits among participants with obesity (Figure 2).

“Weight control” was not associated with any eating habits, with exception of dieting in women with normal weight (*p* < 0.0001, β = 0.79 [0.64, 0.95], Figure 2) and those who were overweight (*p* < 0.0001, β = 0.51 [0.32, 0.71], Figure 2). Psychological symptoms were associated with worse eating habits (e.g., replacing main meals, eating in front of TV/tablet/cellphone, and snacking) in all groups. Of relevance, disordered eating attitudes were strongly associated with dieting among participants with normal weight (*p* < 0.0001, β = 5.28 [3.58, 6.99], Figure 2) and overweight (*p* < 0.001, β = 5.34 [2.54, 8.15], Figure 2). Overall, associations were similar between groups in terms of magnitude and direction, although some of them did not reach statistical significance in overweight and obesity groups, which had a smaller sample size.

## Discussion

The main findings of this study were that: (*i)* eating habits changed due to the COVID-19 quarantine in Brazil, however, these differences were not impacted by BMI, apart from a more frequent report of “replacing main meals with snacks” in overweight vs. normal weight women; (*ii)* determinants of food choice differed as a function of BMI; (*iii)* determinants of food choice and psychological symptoms were associated with eating habits, with some of these associations being affected by BMI.

The COVID-19 pandemic and the set of social distancing measures adopted around the world have resulted in dramatic changes in daily living and lifestyle. A growing number of studies have shown that diet has also changed during this period in several countries (17–21) as well as food choice determinants (22). Our study showed that unlike other studies (23, 24), changes in eating habits during the COVID-19 pandemic did not appear to be influenced by BMI. However, our findings add to the literature by showing that food choice determinants considerably differed across BMI categories.

During the pandemic, “participation in grocery shopping” reduced, whereas “cooking” and “use of delivery service” increased. Regarding the latter, our data seem to corroborate the increased online food purchase seen during pandemic (18, 25, 26), which may be related to the temporary closures of restaurants and bar as well as with a large number of people staying home due to the fear of being infected (26, 27). The number of participants reporting the habit of “snacking” and “eating at table” also increased, while the number of participants reporting the habit of “dieting” decreased. Reductions in dieting could be an attempt to cope with the emotional stress related to the social isolation due to the pandemic, since restrictive diets are associated with emotion regulation difficulties (28). Importantly, and in contrast to our hypothesis, the adaptations in eating habits were overall comparable between women with normal weight, overweight and obesity. Conversely, we observed that food choice determinants that may potentially influence eating habits differed between women with obesity/overweight and those with normal weight. For instance, women with obesity/overweight, as compared with their peers with normal weight, preferentially chose foods to deal with their emotions (e.g., “affect regulation”) rather than being guided by physiological signs (e.g., “need and hunger” and “health concerns”). It has been reported that food choices and preferences may differ in women with obesity and overweight as compared with those with normal weight (4). As women with overweight and obesity commonly use food more often as a mechanism to cope with periods of stress, depression or boredom (4), one could expect that BMI would be a factor influencing eating habits changes during the COVID-19 pandemic. In fact, increased stress is a common psychological reaction to a pandemic situation (29), and individuals with obesity may be more susceptible to stress-induced eating compared to their normal weight peers (23, 30, 31), a notion that seems to be corroborated by this study.

However, these food choice determinants in isolation may have not been sufficient to modify eating habits. It is important to bear in mind that eating habits are driven by a complex, multifactorial process that involves a variety of behavioral and biological aspects (6). In this study, food choice determinants associated with social and economic factors (e.g., “price”, “convenience”, “sociability”, “social norms”, “social image”) did not differ as a function of BMI. This could partially explain the absence of major differences in eating habits during the pandemic between women with obesity/overweight and those with normal weight.

Emotional eating has been shown to be associated with multiple psychological symptoms, such as depression, anxiety, and stress (32). In the present study, we found positive associations in all groups between psychological symptoms and unhealthy eating habits, such as eating in front of the TV, replacing main meals with snacks, and snacking, which are habits closely related to emotional eating. Conversely, psychological symptoms were negatively associated with healthier eating habits, such as eating at the table. Interestingly, despite emotional eating being demonstrated to be more prevalent amongst overweight/obese individuals during the COVID-19 pandemic (23, 31), our data suggest that the effects of the pandemic on emotional health were comparable between different BMI, indicating the indistinct need for nutritional support during this period, regardless of the individual's body weight. One may speculate that the dissonance between studies may be, at least partially, related to cultural, socio-economical, public-health (e.g., access to health support), and severity of the pandemic between countries assessed. Nutritional counseling during a pandemic is recommended, considering that healthy eating habits are possibly important to maintain a healthy immune system (33, 34), and that eating disorders may worse their symptomatology during COVID-19 pandemic (35). Nutritional counseling is also relevant to ensure that pandemic and quarantines do not inflate obesity rates as a consequence of inappropriate eating habits (36).

Our study is strengthened by the fact that data was collected during the most severe period of the pandemic in Brazil, thus representing the most restrictive stay-at-home orders. In addition, our data add to the existing, though limited, literature on the topic by examining the influence of BMI on potential modifications to eating habits and food choice determinants. However, this study is not without limitation. The use of self-reported questionnaires may lead to some degree of imprecision on data reporting. Also, the lack of previous validation for the eating habits assessment instrument may be perceived as a limitation. Importantly, to the best of our knowledge, there is no validated instrument to directly assess eating habits available in the literature. Therefore, we based our questions on the Dietary Guidelines for the Brazilian Population (7), to better capture the most relevant eating habits of our sample. The use of and self-reported weight and height may also be perceived as a limitation of the study, thus warranting further studies using objectively-measured data; however, self-reported weight and height measurement were previously found to be a valid measure in men and women across different BMIs (37, 38). The lack of direct assessment of socioeconomic status may be seen as limitation; however, we did assess educational level, and though and not a direct measure, it can be used as a proxy of socioeconomic status (39, 40). Finally, ours was a convenience sample, and predominantly comprised white eutrophic women who had a university degree, which is not fully representative of the entire Brazilian population, thus limiting the generalizability of the present findings and warranting further studies with large, more heterogeneous samples, and including qualitative research for a more in-depth understanding of the problem.

In conclusion, eating habits were modified during the COVID-19 outbreak among Brazilian women, irrespective of BMI. Nonetheless, determinants of food choice differed between BMI categories. Finally, we showed that, during the COVID-19 quarantine, determinants of food choice and psychological symptoms were associated with eating habits. The understanding of new eating habits and food choice determinants emerging from the COVID-19 pandemic may help tailor better policies and interventions focused on preventing the risks of food insecurity and overweight in specific populations.

## Data Availability Statement

The raw data supporting the conclusions of this article will be made available by the authors, without undue reservation.

## Ethics Statement

The studies involving human participants were reviewed and approved by Ethics Committee for the Analysis of Research Projects of Clinical Hospital of FMUSP, Presentation Certificate for Ethical Appreciation number 33561720.2.0000.0068. The patients/participants provided their written informed consent to participate in this study.

## Author Contributions

BM and FS: Conceptualization, investigation, writing–original draft, visualization, project administration, formal analysis, funding acquisition. GE: Investigation, formal analysis, writing–review, editing, funding acquisition. HS: Investigation, formal analysis, writing–review, editing. MA, DC, and KC: Investigation, formal analysis, writing–review, editing. FB and HR: Writing–review, editing. AP: Formal analysis, visualization, writing–original draft, funding acquisition. BG: Conceptualization, writing–original draft, supervision, funding acquisition. CN: Conceptualization, investigation, writing–review, editing, funding acquisition, project administration, supervision. All authors contributed to the article and approved the submitted version.

## Conflict of Interest

The authors declare that the research was conducted in the absence of any commercial or financial relationships that could be construed as a potential conflict of interest.
